# An expeditious green route toward 2-aryl-4-phenyl-1*H*-imidazoles

**DOI:** 10.1186/s13588-014-0009-7

**Published:** 2014-09-20

**Authors:** Debasish Bandyopadhyay, Lauren C Smith, Daniel R Garcia, Ram N Yadav, Bimal K Banik

**Affiliations:** 1 Department of Chemistry, The University of Texas-Pan American, 1201 West University Drive, Edinburg 78539, TX, USA

**Keywords:** Imidazole, Green chemistry, Ultrasound, Heterocycles, Medicinal chemistry, Azaheterocycles

## Abstract

**Background:**

Azaheterocycles are an important class of compounds because of their highly potent medicinal activities, and the imidazole subcategory is of special interest in regard to drug discovery research.

**Findings:**

An expeditious synthetic protocol of 2-aryl-4-phenyl-1*H*-imidazoles has been accomplished by reacting phenylglyoxal monohydrate, ammonium acetate, and aldehyde under sonication. Following this green approach a series of 2-aryl-4-phenyl-1*H*-imidazoles has been synthesized using diversely substituted aldehydes.

**Conclusions:**

A rapid and simple synthetic procedure to synthesize diversely substituted 2-aryl-4-phenyl-1*H*-imidazoles has been reported. Other salient features of this protocol include milder conditions, atom-economy, easy extraction, and minimum wastes. The present procedure may find application in the synthesis of biologically active molecules.

**Graphical Abstract:**
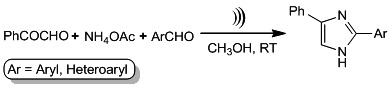
An expeditious synthetic protocol of 2-aryl-4-phenyl-1*H*-imidazoles has been accomplished by reacting phenylglyoxal monohydrate, ammonium acetate, and diversely substituted aldehydes under sonication.

## Findings

Global safety of nature is one of the major criteria in modern science and technology, and the concept of 'green chemistry' has been universally adopted to protect human health and environment. Since the last decade of the twentieth century, protection of environment has been considered as one of the major issues by the chemical scientists and R and D experts [1]. The most significant way to fulfill this eco-requirement is to avoid or reduce the use of hazardous solvents and toxic chemicals and to develop new reactions which can minimize unnecessary formation of the by-products (wastes). Development of such methodologies can provide substantial contribution to green chemistry [2],[3]. Accordingly, development of greener methods has become a significant and prevalent research topic at present age. In synthetic chemistry, the effort has been made on the development of alternative synthetic routes to undertake the desired chemical conversions with nominal exposure of toxic wastes to the environment. Synthesis of desired molecules by sonication is regarded as a substantial green approach [4],[5] to protect the environment by minimizing chemical hazards. Recent research on synthetic organic chemistry indicates that ultrasound can be used as an important device to achieve a number of chemical reactions in high yield and within a shorter reaction time [6]. On other hand, imidazole pharmacophore is present in several pharmacologically active organic molecules including natural products. For example, imidazole scaffold is present in the benzodiazepine antagonist flumazenil, amino acid histidine, the antiulcerative agent cimetidine, the hypnotic agent etomidate, the proton pump inhibitor omeprazole, and so on [7]. Subsequently, there is a continuous need to develop concise and rapid method for the preparation of biologically important and medicinally active imidazole derivatives.

## Results and discussion

Our laboratory is engaged in the green synthesis of novel pharmacophores for many years. To fulfill this goal, we have extensively used automated microwave and ultrasound to induce green technologies in chemical synthesis. We have successfully applied microwave technology to synthesize many novel pharmacophores including, but are not limited to, stereoselective synthesis of ?-lactams [8], *N*-polyaromatic pyrroles [9],[10], anticancer quinoxalines [11],[12], pyrrole-bearing ?-lactams [13]-[16], 1,4-dihydropyridines [17], ?-aminophosphonates [18], and so on.

**Scheme 1:**
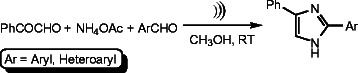
**Ultrasound-assisted synthesis of 2-aryl-4-phenyl-1** ***H*-imidazoles.**

Ultrasound-promoted synthesis of novel anticancer pyrroles has been reported from our laboratory [19]. It has been discovered that ultrasound-assisted aza-Michael reaction proceeds much faster in water than in organic solvents or solvent-free condition without using any catalysts or supports [20]. Because of the importance of 2-aryl-4-phenyl-1*H*-imidazoles in medicinal chemistry, we envisioned the development of an efficient and rapid procedure for the synthesis of 2-aryl-4-phenyl-1*H*-imidazoles without using any catalyst or support is timely and highly challenging. Following our newly developed procedure, a series of 2-aryl-4-phenyl-1*H*-imidazoles was prepared (Scheme 1) under ultrasonic irradiation at room temperature without using any catalyst or support.

Our ultrasound-induced reaction (Scheme 1) has been tested with diversely substituted aldehydes. The results have been summarized in Table 1. No catalysts were necessary for the progress of the reaction. All the ultrasound-assisted reactions were very rapid and completed within an hour to give the desired products in moderate to good yields (57% to 73%).

**Table 1:** **Ultrasound-mediated synthesis of 2-aryl-4-phenyl-1** ***H*-imidazoles**

**Entry**	**Product**	**Time (min)**	**Yield (%)**^**a**^
1		25	66
2		35	59
3		30	63
4		40	64
5		55	61
6		60	57
7		35	61
8		25	69
9		30	73

^a^Isolated yield.

Apparently, the presence of electron-donating or electron-withdrawing substituents has no significant effect on the reaction. It has been reported by Zuliani *et al.* that the reaction proceeds through hemiacetal formation in the presence of methanol [7]. The introduction of ultrasound (i.e., sound energy with frequencies in the range 15 kHz to 1 MHz) into liquid reaction mixtures is known to cause a variety of chemical transformations. Ultrasonic irradiation of liquid reaction mixtures induces electro hydraulic cavitations by which the radii of preexisting gas cavities in the liquid oscillate in a periodically changing pressure. These oscillations eventually become unstable, forcing violent implosion of the gas bubbles. The rapid implosion of a gaseous cavity is accompanied by adiabatic heating of the vapor phase of the bubble, yielding localized and transient high temperatures and pressures. Thus, the apparent chemical effects in liquid reaction media are either direct or indirect consequences of these extreme conditions [20]. The polar solvent methanol has a permanent dipole moment, which allows the coupling between the oscillating electric field and the molecular tumbling to occur with high efficient heating.

### Experimental

#### General procedure for the synthesis of 2-aryl-4-phenyl-1H-imidazoles

A solution of the aldehyde (1 mmol) and threefold excess of ammonium acetate (3 mmol) in methanol (2 mL) was placed in a B5510-DTH (Branson ultrasonic cleaner; Model-5510, frequency 42 KHz with an output power 135 Watts; Branson Ultrasonics, Danbury, CT, USA) sonicator at room temperature. The ultrasonic irradiation was started and a solution of phenylglyoxal monohydrate (1 mmol) in methanol (1 mL) was slowly added dropwise (by a syringe) to the above solution during a period of 15 min. The resulting mixture was continued to irradiate as specified in Table 1. After completion of the reaction (monitored by TLC with an interval of 5 min), the methanol was evaporated under reduced pressure and the crude mass was extracted with ethyl acetate (2?×?5 mL). The combined organic layer was washed with brine (10 mL) and water (10 mL) successively and dried over anhydrous sodium sulfate. The extract was then concentrated, and the crude product was purified using flash chromatography (neutral alumina, 1% triethylamine in methanol) to afford pure compounds.

## Conclusions

In conclusion, the present method demonstrates an operationally simple ultrasound-assisted cleaner procedure for the synthesis of 2-aryl-4-phenyl-1*H*-imidazoles without using any catalyst/solid support. The present economical method satisfies many green chemistry principles such as cost effectiveness, low toxicity, devoid the use of halogenated solvents, atom economy, and most importantly, time. The moderate to good yields of the desired products within shorter reaction time make this methodology a valid contribution to the existing processes in the same field, and their expeditious synthesis as described herein will find wide applications in drug discovery research.

## Competing interests

The authors declare that they have no competing interests.

## Authors¿ information

Lauren C Smith and Daniel R Garcia are undergraduate research participants.
